# What is successful integration in primary health care: qualitative insights from the Chinese public

**DOI:** 10.1080/16549716.2024.2430811

**Published:** 2024-11-19

**Authors:** Jinnan Zhang, Rebecca Mitchell, Ruixue Zhao, Mengyao Li, Wenhua Wang

**Affiliations:** a School of Public Policy and Administration, Xi’an Jiaotong University, Xi’an, PR China; b Health and Wellbeing Research Unit (HoWRU), Macquarie Business School, Macquarie University, Sydney, Australia; c Newcastle Business School, The University of Newcastle, Newcastle, Australia

**Keywords:** Integrated care, people-centered, primary health care, public preference, qualitative study

## Abstract

**Background:**

China is transforming its hospital-centric service delivery system into a people-centered integrated care model, with service delivery organized around the health needs and expectations of people.

**Objective:**

To guide reforms and align with public expectations, this study profiles successful integration in primary health care from the public perspective.

**Methods:**

Guided by the rainbow model of integrated care, semi-structured interviews were conducted in six provinces in China. A total of 58 interviewees completed the interviews. Tape-based analysis was used to produce narrative summaries. Researchers listened to the recordings and summarized by 30-s segments. Thematic analysis was performed on summaries to identify thematic families.

**Results:**

Five themes and 16 sub-themes were generated. Respondents’ expectations were primarily on three themes: clinical integration (such as interaction between professional and client, continuity, and empowering and engaging individuals), functional integration (such as resources management, quality improvement, and reforming payment systems), and system integration (such as institutional distribution and supervision). Yet a few interviewees mentioned professional integration (multi-disciplinary collaboration) and organizational integration (inter-organizational strategy).

**Conclusions:**

Qualitative data were used to reveal public perceptions of successful primary health care integration. Service processes, institutional distribution, regulation, resource management, and quality improvement are more visible to the public and will be priorities for future efforts. Whereas inter-organizational strategies and multi-disciplinary collaboration have been shown to facilitate service improvements. Future efforts could consider how policy efforts can be grounded in visible service delivery through management practices.

## Paper Context

**Main findings:**

The views of the Chinese public for successful integration in primary health care were summarized at five themes and 16 sub-themes.

**Added knowledge:**

In the process of integrating healthcare services, the often overlooked yet crucial perspective of the demand side was elucidated for the first time in the primary care setting in China.

**Global health impact for policy and action:**

Future integration efforts could benefit from soliciting more input from stakeholders and considering how policy efforts can be grounded in visible service delivery through management practices.

## Background

Following healthcare reform, China significantly invested in medical infrastructure, basic public health service equalization, universal health insurance coverage, and essential drug system improvements. These efforts preliminarily enhanced healthcare accessibility and equity [1]. By 2021, Chinese residents’ average life expectancy reached 78.2, aligning with the health levels of middle-income countries [2]. However, China gradually faces an aging population, rising chronic disease burden, and heightened expectations for quality healthcare. By the end of 2022, China’s elderly population aged 60 and above reached about 280 million, accounting for 19.8% of the total population [3]. Factors like aging, lifestyle changes, urbanization, and dietary shifts have increased chronic disease prevalence from 157.4 to 342.9 per 1,000 between 2008 and 2018 [4]. The challenge of managing chronic diseases, alongside growing health awareness, has heightened demand for diverse, quality healthcare services.

Global health system reforms identified primary health care (PHC) as a pivotal element, crucial for promoting individual and community health and reducing economic losses [5–10]. Recent initiatives have also prioritized PHC and quality of care in healthcare reforms globally [11–15]. Consequently, developing a high-quality PHC system is a primary goal for many countries, including China. China’s PHC-based service delivery system, established in the 1950s to 1970s, shifted to a hospital-centric model following the 1980s’ market reforms. The latter has led to higher medical expenses and increased patient complaints, including overmedication, long waiting times, rushed consultations, lack of effective communication, healthcare provider indifference, and perceived lack of effort [16].

Over the past few years, many countries have implemented PHC-based health system integration reforms, showing positive health outcomes and service utilization impacts [17,18]. Integrated care implementation varies globally, influenced by local healthcare policies, economic conditions, and cultural factors [19]. To address the above challenges, China is reorienting its system towards a WHO and World Bank-proposed people-centered integrated care (PCIC) model [16]. People-centered health services adopt a delivery approach that integrates the perspectives of individuals, families, and communities as active participants and beneficiaries, responding to their needs and preferences [20]. Integrated health services ensure access to a continuum of health promotion, disease prevention, diagnosis, treatment, and rehabilitation, catering to individual needs across various system levels and throughout their life [20]. Recently, China introduced policies to strengthen PHC and health services integration, including standardizing community health centers, implementing hierarchical medical systems, and developing family doctor systems [1,16,21,22]. These efforts have resulted in some progress in the accessibility and quality of PHC [16]. Yet, public confidence in PHC remained low, with a marked preference for hospital care, leading to increased hospital use and reduced PHC utilization [21,23,24]. Challenges persist in effectively integrating PHC. For instance, local governments and health care providers lack incentives to promote integration and reshape the health care system. In addition, low salaries and inadequate incentives in PHC facilities make it difficult to attract and retain qualified health care workers. There is also a lack of understanding of the health needs of community residents and their expectations of the health care delivery system [16].

Most new programs, like integrated health services, were government-driven ‘top-down’ initiatives, often overlooking public preferences [25]. Yet, what has been agreed upon is that health decisions should be customized to the needs and values of the public [15,26]. Addressing PCIC model challenges should start with understanding and measuring success indicators in PHC integration from the public’s viewpoint, grounded in people-centeredness. Qualitative research methods are instrumental in exploring and analyzing public perspectives and preferences in healthcare [27]. To guide reforms and meet public expectations, this study aims to profile the successful integration in PHC from the public’s perspective through qualitative research. The insights can inform the development and implementation of PCIC models that meet the specific needs and expectations of the Chinese population. By integrating individuals’ perspectives into healthcare reforms, it is possible to enhance quality of care, improve health outcomes, and promote patient experience in the Chinese healthcare system.

## Methods

### Theoretical framework

Drawing upon the rainbow model of integrated care (RMIC) and the WHO’s strategies for integrated people-centred health services [26,28], we developed a conceptual framework for assessing successful integration in PHC. This framework encompasses six key domains: clinical integration, professional integration, organizational integration, system integration, functional integration, and normative integration. As the primary framework for our study, the RMIC model integrated various existing integrated care models and has been shown to be well applied to explore the integration perspectives of different stakeholders in different countries, including four types of integration processes (clinical integration, professional integration, organizational integration, and system integration) and two enablers for integration (functional integration and normative integration) [28]. WHO’s five strategies for integrated people-centred health services were used to enrich our theoretical framework for providing detailed policy options and interventions for countries and regions with health service integration needs, including empowering and engaging people and communities, strengthening governance and accountability, reorienting the model of care, coordinating services within and across sectors, and creating an enabling environment [26]. This framework informed our interview guide and underpinned the thematic analysis in our research.

### Study design

A qualitative study using semi-structured interviews was conducted to examine the public’s views and expectations for successful integration in PHC. The interview guideline, based on the framework mentioned above, was refined through a pilot study with 15 primary care users in August 2020 and further expert discussions for relevance and appropriateness.

Finally, the study was completed among 58 primary care users from six provinces (one city in each province), including central region (Luoyang City in Henan Province, Taiyuan City in Shanxi Province and Harbin City in Heilongjiang Province); eastern region (Jinan City in Shandong Province and Hangzhou City in Zhejiang Province); and western region (Xi’an City in Shaanxi Province).

### Participants recruitment

Initially, we planned to recruit 8–12 participants from each city, a sample size sufficient to identify common themes across the population [29]. Eligibility criteria included participants aged 18–80, without cognitive impairments, who had visited a PHC organization in the past 12 months. To ensure participant diversity, we paid close attention to achieving a balanced representation across different genders, ages, educational backgrounds, types of health insurance, and chronic disease conditions during the recruitment process. This approach ensured that our study encompassed a wide range of perspectives and experiences, enhancing the validity and applicability of our findings. Recruitment was facilitated by local government officials who assisted in disseminating study information, screening, identifying and contacting eligible participants through community health centers, organizing the interviews, and ensuring participant diversity.

### Data collection

Two researchers (J.Z. and R.Z.) conducted semi-structured interviews from 2 April to 15 December 2021, using a tested open-ended qualitative interview guide. J.Z. and R.Z. are experienced researchers with backgrounds in health policy and management, having previously conducted multiple projects related to PHC. Both researchers received comprehensive training in interviewing techniques and qualitative analysis to ensure consistent and neutral data collection and interpretation. To minimize the potential influence of preconceived ideas, the interview guide was carefully designed to facilitate open-ended responses and to avoid leading questions. During the interviews, interviewers first asked participants to recall their past healthcare experiences and previous PHC visits as a ‘warm-up’. Then, participants were encouraged to express their views and expectations for PHC integration in their own words, using themes from our literature review in the interview guide to facilitate the discussion. The interviewers allowed for free discussion, using the guide merely to prompt exploration of specific areas. If participants made ambiguous statements, the interviewers would clarify and confirm their viewpoints to ensure accurate understanding. This flexible approach allows exploration of interviewees’ values and meanings without imposing preconceived notions [30]. Interviews were face-to-face, one-on-one, lasting 30–60 minutes, conducted in local community centers. All semi-structured interviews were audio-recorded with interviewees’ consent. Immediately after each interview, the interviewer took debriefing notes to capture contextual details, including non-verbal cues and the interview process [31]. Saturation was achieved when new data became redundant, indicating no new information was emerging. For instance, reaching saturation was indicated by repetitive comments during interviews [32]. In total, 58 interviewees from Henan (8), Shandong (11), Zhejiang (11), Shaanxi (8), Shanxi (8) and Heilongjiang (12) provinces completed the recorded interview. Interview recordings were uploaded into NVivo12 software.

### Data analysis

Thematic analysis was used to analyse the interview data [33]. Our analysis combined deductive and inductive approaches, focusing on descriptive insights to let original themes emerge. An initial analytical framework was developed based on an extensive literature review [26,28]. Two researchers (J.Z. and R.Z.) independently transcribed the interviews by summarizing every 30-s segment. Parallel to each 30-s segment, three researchers noted themes from the analytic framework or made notes about the behaviour of the interviewee, such as hesitations, lack of comfort, refusal to answers or tangents. The transcriptions were discussed within the research team to ensure faithfully reflecting the interviewees’ thoughts. This transcription method has been tested and used by qualitative researchers [34]. In total, 1,742 narrative summaries were generated. Thematic analysis was then applied to narrative summaries to identify thematic families. Initially, two analysts (J.Z. and R.Z.) independently produced codes until the coding became stable and consistent. The analysts then met to resolve differences and refine code definitions and structure in discussion with the research team. When coding stabilized, one analyst (J.Z.) continued coding the remaining summaries, later verified by the second analyst (R.Z.) [35]. The analysis produced 67 codes with clear and mutually exclusive definitions.

### Ethics and consent

Ethical approval was granted by the Xi’an Jiaotong University Ethics Committee (number 2020–1344). Written informed consent was obtained from all participants before the semi-structured interviews. Interviewees were informed that participation was voluntary, with data kept confidential and used solely for research purposes. To protect the identifiable information collected from the interviewees, all personal data was anonymized. Each participant was assigned a unique identifier, and any potentially identifying details were removed during transcription. The anonymized data was securely stored on encrypted devices, accessible only to the research team.

## Results

### Participant characteristics

Table 1 summarizes characteristics of 58 community residents from urban China, interviewed one-on-one after visiting community health centers. Half of the participants were female, and an equal percentage had attained a college education or higher. One-fifth were over 60 years old, and a quarter of them had at least one chronic disease. Regarding health insurance, 70% were enrolled in urban employee basic medical insurance – designed for current or former employees – while the remaining participants were covered by urban and rural resident basic medical insurance, catering to the unemployed, students, and children.

**Table 1.** Demographics of participants (*N* = 58).

Characteristic	No. (%)
Age, mean (SD), years	45 (14.3)
<60	47 (81.0)
≥60	11 (19.0)
Sex	
Male	29 (50.0)
Female	29 (50.0)
Education level	
Primary school or below	4 (6.9)
Junior school	10 (17.2)
High school or technical secondary school	14 (24.1)
Undergraduate or junior college	20 (34.5)
Master’s degree or higher	10 (17.2)
Medical insurance	
Urban employee basic medical insurance	41 (70.7)
Urban resident basic medical insurance	17 (29.3)
Chronic diseases status^a^	
Yes	14 (24.1)
No	44 (75.9)

^a^Chronic diseases here only included diabetes and/or hypertension.

### Five themes of successful integration in PHC from public’s perspective

As shown in Table 2, five themes, 16 sub-themes and the typical quotes were generated from the analysis of our interview data. The five themes were clinical integration, professional integration, organizational integration, systems integration, and functional integration. While normative integration in the theoretical framework was not covered by the interview data.

**Table 2.** Five themes, 16 sub-themes and the exemplar quotes of successful integration in PHC from qualitative interviews with 58 PHC users.

Themes	Sub-themes	Exemplar quotes
Clinical integration	Interaction between professional and client	“The patient is already uncomfortable, so I hope the doctor can have a better attitude towards the patient.” (TY4) “During the consultation process, doctors should communicate effectively and patiently with patients. They should not just inform patients of the treatment plan and the medications they need to buy, and then send them off to pay the fees.” (LY6)
	Continuity	“I hope the consultation process can be simpler and not as complicated as in hospitals.” (HRB9) “Because I have a chronic disease and often need to go to the community health center to buy regular medication, I hope that professionals who are very familiar with my condition will manage my illness on a long-term and consistent basis.” (JN11) “I hope that the doctor will keep the patient’s phone number and contact them a few days later to ask if they are taking the medication on time and if their condition has improved.” (HRB8)
	Empowering and engaging individuals	“I hope that professionals can provide patients with daily life tips to promote health through diet, exercise, and other methods, while avoiding medication as much as possible.” (JN4) “I hope home health services can be offered to elderly people living alone in the community, with an increased number of staff dedicated to this service.” (TY2)
	Service scope	“I hope the scope of health services can be more comprehensive, as a community health center serve a wide range of clients, including young people, the elderly, and children in the community.” (HRB1)
	Coordinating care for individuals	“If the community health center cannot cope with the situation, it should inform the patient directly and help him or her to go to a more specialised hospital.” (JN8)
	Accessibility	“I do remember the community health centers would close in the evenings. I wish there were one or two doctors on duty at the facility at night that could provide basic services to patients.” (HRB7)
Professional integration	Multi-disciplinary collaboration	“Departments in institutions should cooperate with each other to provide patients treatment plans.” (TY8)
Organizational integration	Inter-organizational strategy	“Specialists from hospitals should come to community health centers regularly to train general practitioners.” (JN4)
System integration	Allocation of primary care institutions	“Community health centers must be built in regions with dense residential populations.” (XA5)
	Supervision and management	“The health system should supervise and manage the community health centers, including the environment, personnel, and equipment.” (HZ6)
	Governance for public and private sectors	“Public and private institutions can play roles in primary care together, as they both have their own strengths.” (TY2)
	Gatekeeping	“When the disease is not serious, the patient should first go to the community health center for a preliminary diagnosis.” (LY8)
Functional integration	Resources management	“A good community health center should first have a certain number of doctors and nurses.” (LY8) “I feel that the government should now actively support the development of community health centers by investing in equipment and so on. This will ease the pressure on hospitals and also facilitate the residents’ access to health care.” (JN5)
	Striving for quality improvement and safety	“Community health centers should continue to improve treatment effectiveness.” (HRB11) “Doctors should consider various factors to make an accurate diagnosis and try to avoid diagnostic errors.” (HRB7)
	Improving funding and reforming payment systems	I hope the cost at the community health center would not be too high or the people can’t afford it. (HRB12) I hope the drugs in the community health center can be covered by health insurance (HZ1)
	Information management	“The patient’s medical records should be saved and shared among all community health centers within the same city. It is unacceptable to me that my previous visit information will not be available when I go to another community health center next time.” (TY7)

Clinical integration included six attributes: 1) interaction between professional and client; 2) continuity; 3) empowering and engaging individuals; 4) service scope; 5) coordinating care for individuals; 6) accessibility. For interaction between professional and client, the common expectations were that professionals pay attention to the patients’ psychological needs (including showing good attitudes, caring for patients, and respecting their privacy) and communicate effectively with them (including exploring disease-related information through detailed questioning and providing clarity to patients). For continuity, participants expected the service process to be simple and smooth, the service to be provided by familiar professionals, and there to be follow-up after consultation. For empowering and engaging individuals, the common expectations were that residents could be strengthened in their ability to self-care by receiving health-related knowledge, that they could be informed and share decision-making with professionals in the service process, and that unserved and marginalized individuals could be reached (including remote or in-home services for mobility impaired groups, easy-to-understand services for limited comprehension groups, basic medical care to low-income groups). In addition, common expectations at this level also included PHC facilities to extend their business hours, to provide coordinated services (upward referrals), and to provide comprehensive services, including clinical services, preventive services (health check-ups, vaccinations), and rehabilitation services.

Functional integration included four attributes: 1) resource management; 2) striving for quality improvement and safety; 3) improving funding and reforming payment systems; 4) information management. For functional integration, the common expectations were that organizational resources (including human resources, medical equipment, and medicines) were well allocated, service quality (including effectiveness, diagnostic accuracy, safety, efficiency, and patient experience) was continuously improved, and information technology (including electronic medical record system and online registration system) was appropriately used.

System integration included four attributes: 1) allocation of primary care institutions; 2) supervision and management; 3) governance for public and private sectors; 4) gatekeeping. For system integration, the common expectations were that the PHC facilities should be the first contact point for patients, and their location, number, and scale should be planned based on the population size and health needs in the region. Public and private institutions should be properly governed according to their respective characteristics. In addition, there should be a uniform regulatory framework (including hygienic conditions, medical qualifications, standards of practice) to regulate PHC providers.

Professional integration included one attribute: multi-disciplinary collaboration. The public expected the different departments in PHC organizations could collaborate to provide comprehensive health care services to patients.

Organizational integration included one attribute: inter-organizational strategy. The common public expectation was that PHC organizations should closely collaborate with hospitals, such as operational collaboration or medical training.

## Discussion

Through semi-structured interviews with PHC users across six provinces, our study revealed the public’s view on successful PHC integration, identifying five main themes and 16 sub-themes. The most emphasized areas by the public were clinical and functional integration, highlighting a demand for PHC that is comprehensive, continuous, coordinated, accessible, affordable, and quality-focused, underpinned by well-allocated resources and professional interactions. Conversely, areas such as professional and normative integration, despite their known benefits to quality, were seldom mentioned by participants.

Clinical integration refers to the degree of consistency and coordination in the process of care delivery to individual patients. It emerged as the theme with the richest content in our findings, as it could be perceived directly by patients during the consultation process [18,36]. Interaction between professional and client, a sub-theme of clinical integration, forms the cornerstone of health service delivery in integrated care. Preferences for effective communication and attention to psychological needs in professional-client interactions, supported by prior evidence [37–45], reflected public expectations for emotional support, perceived competence, and respect [46–48]. Professionals providing emotional support and empathy could reduce patients’ anxiety and uncertainty [46]. Patients perceived professionals demonstrating positive attitudes and behaviors as more competent and trustworthy, leading to improved adherence to treatment plans and health outcomes [48]. Furthermore, when professionals demonstrated attentive and respectful behavior, patients would feel respected and validated, which helps cultivate a sense of autonomy and empowerment in the professional-client relationship [47].

Empowering and engaging individuals, another sub-theme of clinical integration, is a key strategy among the WHO’s five approaches to integrated people-centred health services [26]. Existing research suggested that receiving health education allowed patients to increase health knowledge, improve self-management skills, and actively participate in decision-making [49–51]. Reaching the underserved and marginalized is critical not only to ensuring universal access to quality care but also to achieving broader social goals such as equity, social justice, and solidarity, thereby helping to build social cohesion [26]. In recent years, China has made significant policy efforts to address the healthcare needs of underserved and marginalized populations such as establishing 14 basic public health services, improving PHC services in rural areas, and integrating urban resident basic medical insurance and new cooperative medical scheme into urban-rural resident basic medical insurance [1,21].

The other four sub-themes within clinical integration, including continuity, service scope, coordinating care for individuals, as well as accessibility, also known as the ‘4 Cs’ of PHC termed by Barbara Starfield, were essential components of any successful PHC system [8]. For continuity, three dimensions including information continuity, relationship continuity, and management continuity were all mentioned by the population. National policy efforts have been made to improve continuity, such as building family doctor contract service model and tiered health-care delivery system with bidirectional referral mechanisms [52,53]. In terms of service scope, the expected PHC services, including clinical, public health, and rehabilitation services, have been essentially covered by the existing service scope of China’s PHC institutions [53]. The next focus should be on how to improve the quality of these services in practice. As for accessibility, achieving it requires system integration, including strategic geographical distribution of PHC institutions. Besides, it is widely accepted that care coordination across all health care providers and related disciplines will improve the quality of care and thus meet the preferences of the patients involved [54]. The coordinated experience of services provided to individuals needs to be achieved through professional integration such as multidisciplinary collaboration and organizational integration such as referral systems.

In comparison, there was relatively little content in the interview data that can be categorized under the theme of professional integration, which differs from findings in other countries [37]. This discrepancy may stem from a mismatch between the perceived complexity of multidisciplinary care and the goal of treating general illnesses in China’s PHC. Multidisciplinary collaboration, a key integrated care strategy, has effectively improved patient health, service utilization, satisfaction, and reduced costs in developed countries such as the US, Australia, Canada, and the Netherlands [55,56]. Since 2016, Chinese primary care has employed a multidisciplinary collaboration among general practitioners, nurses, and public health doctors to serve community residents [52]. However, the ideal multidisciplinary team from the patient’s perspective, where professionals from different disciplines work together to deliver services, was rarely seen in the Chinese primary care scene. The reality was that more multidisciplinary teams were still responsible for their own departments and delivered non-integrated services to patients separately. Improvements could come from clarifying roles and adopting a patient-centered approach to better address patient needs [15]. In addition, information communication technologies (ICT) systems, one of the priorities for functional integration, could be used to support care coordination through information sharing within multidisciplinary teams, which has been widely used in integrated community-based PHC in countries such as Canada and New Zealand [57].

Similar to professional integration, organizational integration has also been largely overlooked, aligning with another study’s findings from China [58]. This could be due to significant challenges in collaboration between hospitals and primary care providers in China, marked by limited cooperation and dominant competition [59]. One study has underscored the healthcare system’s fragmentation, with hospitals and PHC institutions operating as separate entities with distinct goals and priorities [1]. The historical focus on hospital-based care and the healthcare system’s hierarchical structure have led to poor integration and coordination between hospitals and PHC institutions [16]. Moreover, the competitive healthcare landscape in China exacerbated the lack of collaboration. The fee-for-service reimbursement model, encouraging high-volume and profitable procedures, has fostered a competitive environment where hospitals prioritize attracting patients and revenue generation. Conversely, PHC institutions frequently face resource limitations and inadequate funding. This dynamic promoted competition over collaboration, hindering PHC institutions from becoming viable alternatives to hospital care [16]. To tackle these issues, policy initiatives, such as the establishment of integrated care systems and the strengthening of referral mechanisms, have been launched to enhance collaboration and partnership between hospitals and PHC institutions [16]. These initiatives recognized the importance of collaboration in improving healthcare outcomes, reducing costs, and achieving a more people-centered approach.

System integration, a holistic approach centered on people’s needs to serve the population, has been widely discussed [18]. Firstly, the consensus was that the planning of PHC facilities’ location, number, and scale should align with the regional population size and health needs, reflecting the population-based care model of integrated care [18]. This expectation also represented a way to achieve accessibility within clinical integration. To meet the needs of the population, the Chinese government has invested heavily in increasing the number of PHC facilities over the past decade [21]. Additionally, the government has set specific location requirements for these institutions. A 2017 national policy mandated that PHC facilities be located to ensure service access within 30 min for their target population, aiming to enhance accessibility [60]. Second, the public advocated for a unified regulatory framework for PHC providers. Such a framework could promote the integration of services, improve quality and standardization, enhance information sharing, support workforce development, and ensure financial sustainability [1,22]. Additionally, the public saw governance that considers the unique aspects of differently owned PHC providers as essential for integration. In China, more than half of PHC facilities were publicly owned [4]. Yet limited evidence indicated that public PHC facilities outperform private ones [61]. Different ownerships of primary care facilities offered unique advantages in accessibility, coordination, comprehensiveness, and continuity [61–65]. The focus could be on how to optimize the operational efficiency of existing institutions while ensuring the public benefit. Furthermore, based on existing evidence, exploring public–private partnerships in PHC as crucial tools for achieving universal health coverage could be considered [66].

Functional integration was another rich theme. It involved linking funding, information, and management to maximize the system’s overall value [67]. Among them, resource management emerged as a key focus, with the public expecting rational allocation of personnel, medical equipment, and medicines in PHC organizations based on patient needs. Substantial evidence indicated that gaps in health resources hinder care delivery and access, leading patients to bypass facilities lacking necessary resources [68–70]. However, untargeted overinvestment has not effectively improved PHC performance, as shown in various national and regional systems [71]. Over the past decade, China has invested heavily in building well-equipped PHC organizations, including infrastructure development, training and recruitment of PHC professionals, and medical equipment procurement [21,22]. Nevertheless, patients have not been attracted to well-equipped but low-quality PHC facilities [72]. This explained the public’s call for rational and targeted resource investment in PHC facilities. From an integration standpoint, resource investment strategies in PHC should focus more on the institution’s practice characteristics, funding arrangements, and their alignment with policy goals and patients’ well-being [71]. Quality improvement mechanisms, another sub-theme within functional integration, have also been shown to be a key element of high-performing health care organizations [61,73]. Additionally, the development of information systems played a key role in providing continuous and coordinated services to patients. However, health information system development in China is still nascent. Numerous issues persisted, including the separate operation of the resident health records and electronic medical records in PHC, and challenges in electronic linking and information transfer between PHC organizations and hospitals [21].

We did not yield much information from interview data regarding normative integration, another driver of integration apart from functional integration. It was not surprising because normative integration such as shared vision, visionary leadership, and linking cultures was hard to quantify and perceive. However, such informal coordination mechanisms based on shared values among individuals, professionals, and organizations were thought to have the potential to influence professional behavior and attitudes and consequently integration [18]. For instance, organizational culture was increasingly recognized as a crucial determinant in implementing evidence-based practices in healthcare settings [74–76]. Recently, organizational culture has been identified as one of the key organizational factors in the context and capabilities for integrating care framework [77]. Other features of normative integration, like leadership, cohesive mission, and senior manager support have also been shown to be associated with higher hospital performance [73,78,79]. All the above normative integrations could be potential breakthroughs in the health care integration process when structural inputs fail to make progress.

One of the strengths of our study is the diversity of the sample, which includes participants from various regions of China, different genders, age groups, and types of medical insurance. Additionally, we utilized an established analytical framework, the RMIC, developed through a comprehensive review of the literature on integrated care models. This scientific framework allowed us to systematically explore the structure of integrated care while considering the context of PHC institutions in China. However, there are several limitations to our study. Although our sample included diverse PHC users, we did not compare preferences for successful PHC integration among users with different characteristics due to space constraints. Future research could focus on specific themes of integration to compare preferences and views across different populations. Additionally, there is a high demand for health care among China’s rural population. Our study did not explore their expectations. Future research could be conducted to investigate rural populations’ preferences for successful integration in PHC and compare them with urban populations.

## Conclusion

Overall, China is optimizing the health service delivery system through building PCIC to address new health challenges. Using the qualitative data, knowledge was generated to reveal the public’s views for successful integration in PHC. Service processes at the micro level, institutional distribution and regulation at the macro level, and resource management and quality improvement are more visible to the public and will be priorities for future efforts. Whereas inter-organizational strategies and multi-disciplinary collaboration at the meso level have been shown to facilitate service improvements. Future efforts could consider how policy efforts can be grounded in visible service delivery through management practices.

## Data Availability

Data underlying this article cannot be shared publicly to protect the privacy of individuals who participated in the study. Aggregate data will be shared on reasonable request to the corresponding author.
